# On the Causes and Treatment of Colds in the Head of Influenzal Origin

**Published:** 1895-05-04

**Authors:** 


### ON THE CAUSES AND TREATMENT OF COLD IN THE HEAD OF INFLUENZAL ORIGIN.

This commonplace and very uninteresting ailment has this year attained to great importance and received unusual attention, for the sufficient reason that the present epidemic of influenza has very generally mimicked the old-fashioned cold in the head, so much so, that many sufferers have been deceived and led to regard as a trivial ailment that which, by repeated exposures to a Siberian severity of weather, has developed into one of the most serious of maladies. This experience illustrates forcibly the truth of the classic saying, "that in times of epidemics all diseases are apt to assume a malignant type." The modern explanation of which phenomenon, I assume, is that the specific germ is grafted on to the ordinary disease? These combinations explain the apparent infinite variety of symptoms observed, and the varying types in different epidemics of influenza. It seems probable that the most fruitful exciting cause of cold in the head is the chilling effect of the absurd headgear that men affect this bleak winter. Where is the sanity in wearing the same head covering whether the temperature is at zero or whether it is 70 degrees Fahrenheit in the shade? Nothing is more unsuitable for the purposes of warmth than a silk hat with a leather lining.

We pride ourselves upon our civilisation, and yet an Esquimaux is in advance of us in the matter of winter head covering. Can we be surprised if neuralgias and cold upon cold, with all their favouring potentialities for grave complication, abound? Most of us have realised that it is folly not to keep our feet warm and dry by means of "Jaeger"-lined boots and rubber snow shoes. But few act upon the elementary principle of personal hygiene, that the head is also an extremity to be kept warm.

Ladies and the working-classes are not suffering so much in this epidemic for the reason, probably, that they wear something warmer than silk or felt hats. It is the professional and official classes who are at first mostly attacked, and thus become foci of infection. As men insist on clinging to their patent of respectability—the silk hat—it is to be wished that it was more generally known that a felt lining adds considerably to the warmth of the head. By the substitution of this simple and inexpensive material for the usual leather one, neuritis of rheumatic origin may be cured or prevented.

The treatment of head cold is a matter of attention to detail, and no symptom is so trivial as to be unworthy of sympathetic attention. A remark frequently heard, and too often justified, is, that "It is useless sending for the doctor, as he will only tell me to keep indoors, and order me a saline draught!" or "Dr. A takes no interest in slight ailments; he has nothing to suggest." The practical form of professional sympathy is the suggestion of remedies.

It used to be said that the cure for the old-fashioned form of influenza was a pint of champagne! There is more than a modicum of truth in the statement, as the nourishment should be both light and stimulating. Another way of emphasising the place of alcohol in the diet sheet is found in the conundrum: What is the key to the treatment of influenza? Answer—The cellar key!

To take the symptoms, briefly, in detail. For the pains generally warmth is the first essential, and that can only be effectually obtained in bed. Cold invariably brings back the pains.

If the headache is severe, a dose of phenacetin, gr. v.—x., may be given; but it seems almost certain that antipyrin and salicylate of soda given as a routine treatment in full doses, defer and prolong the convalescence, increase debility, and favour cardiac collapse. Hot sponges best relieve brow-ache, eye-ache, and the intolerable feeling as of the nasal passages being blocked.

Nasal douching is most comforting and effectual. Macnaughton Jones' nasal tabloids, made by Burroughes and Wellcome may be used. One tabloid in a tumbler of hot water. An ordinary Higginson's syringe acts very well. The lotion so made is both antiseptic and germicidal. The patient is directed to sniff with each jet of the syringe, by which means the nasal passages are effectually cleared. This may be done thrice daily. This nasal douching seems to be both curative, prophylactic, and preventative of complications, and lessens the risk of spreading infection by naso-pharyngeal discharge.

Mastoid abscess, purulent otitis, abscess of the frontal sinuses, catarrhal deafness, loss of sense of smell, and post nasal troubles are rendered less likely to follow. To prevent multiplicity of prescriptions, an effective gargle may be made with the same tabloids.

For the conjunctivitis, an eye lotion made up with ext. opii. liq. ʒi., boracis ʒi., to six ounces of water, to be used with an eye bath, will usually be sufficient. For the general treatment whilst the skin is not acting and the patient is much depressed, the popular remedy of tinct. quininæ am. in ʒi. doses every sixth hour, if given in soda water, sits lightly on the stomach, keeps up the appetite, and relieves the neuralgia; to be substituted by a mineral acid as soon as the temperature is normal or sub-normal. This sometimes falls to 95·8 degrees Fahr. It is well to give a daily aperient from the beginning. Men almost invariably suffer from the multiform discomforts of constipation when put to bed. If the influenza is more of the malarial than catarrhal type, "Warburg's Tincture" is a useful medicine. It cleans the tongue, stimulates the emunctories, is a gentle aperient, and has a tonic rather than a lowering effect. Hot milk and whisky generally agrees well throughout convalescence, to be taken at eleven a.m. and bedtime.

Patients take a rapid turn for the better as soon as change of air is obtained. It seems probable that persons who have once had the disease badly carry about with them for a long period the germs of the disease lying dormant, ready to spring into activity at the first chill taken. A similar fate to that which befalls a patient who has lived in a malarial district. It is a recrudescence, not a fresh attack. For these and their neighbours the only safety lies in the careful avoidance of cold. Cold is the spark that ignites the magazine, the one element lacking to produce an explosion.

How long infection lasts is uncertain; probably as long as there is cough or naso-pharyngeal catarrh. Patients are loth to be convinced that disinfection is necessary, nor will they submit to prolonged isolation, nor, in fact, to any isolation at all.

Yet the public call out for the medical faculty to do some great thing, and will not observe the elementary precautions themselves of avoiding chill, infected persons, nor infected houses.

Is it surprising that the epidemic spreads, when we read that members of Parliament, stricken with influenza, leave their beds, ignoring their doctor's orders, and whilst taking part in the divisions in the House of Commons, scatter the seeds of disease broadcast amongst their colleagues?

